# Door-to-Balloon Time in ST-Elevation Myocardial Infarction (STEMI) Patients Undergoing Primary Angioplasty in Myocardial Infarction (PAMI): An Observational Study From a Tertiary Care Centre

**DOI:** 10.7759/cureus.62222

**Published:** 2024-06-12

**Authors:** Keyur Shah, Sandeep Gore, Vivek Solapure, Pradip Shah, Jishmi K Shah

**Affiliations:** 1 Emergency Medicine, Pramukhswami Medical College, Karamsad, IND; 2 Emergency Medicine, Fortis Hospital Mulund, Mumbai, IND; 3 Emergency Medicine, MGM Hospital Vashi, Navi Mumbai, IND; 4 Internal Medicine, Fortis Hospital Mulund, Mumbai, IND; 5 Anaesthesiology, Pramukhswami Medical College, Karamsad, IND

**Keywords:** service quality, time delay, door-to-balloon time, primary angioplasty, st-elevation myocardial infarction (stemi)

## Abstract

Background

ST-elevation myocardial infarction (STEMI) requires swift intervention, with primary percutaneous coronary intervention (PCI) being essential to limit myocardial damage. The key factor affecting PCI effectiveness is the door-to-balloon (DTB) time. This observational study evaluated DTB times in STEMI patients at a tertiary care center who underwent primary angioplasty, examining adherence to benchmarks and identifying factors contributing to delays.

Methodology

This prospective observational study was conducted from March 2017 to August 2018 at Fortis Hospital Mulund, Mumbai, India. It included 171 STEMI patients aged 18 and older who underwent primary angioplasty. Patients with non-ST elevation myocardial infarction (NSTEMI), those who received thrombolysis, or had medical contraindications to primary angioplasty were excluded. Data on key time intervals were collected via direct observation and then analyzed using SPSS for Windows, Version 15 (Released 2006; SPSS Inc., Chicago, United States). Qualitative data were summarized using frequency and percentages, whereas quantitative data were presented as mean (±SD). T-test was applied to compare the mean duration between the two groups, i.e., DTB time ≤90 minutes and DTB time >90 minutes, and a p-value <0.05 was considered statistically significant.

Results

The participants had a mean age of 56.5 (±13.1) years and were predominantly male (78.4%). The mean DTB time was 70.21 (±29.16) minutes, with 79.5% achieving ≤90 minutes. Patient-related delays (48.6%) were mainly due to consent issues (31.4%), which was the most predominant cause. Hospital-related delays (51.4%) included catheterization laboratory occupancy (14.3%) and diagnostic delays (14.3%). Patients with DTB times >90 minutes had significantly longer durations in all procedural steps except door-to-ECG time.

Conclusion

This study underscores the complex challenges in achieving timely DTB times for STEMI patients undergoing primary angioplasty. Overcoming these barriers through targeted interventions is essential for optimizing management and enhancing outcomes. Insights into delay factors inform evidence-based strategies to improve the timeliness and effectiveness of STEMI care delivery.

## Introduction

ST-elevation myocardial infarction (STEMI) characterizes a critical medical emergency demanding rapid and precise intervention to mitigate myocardial damage and enhance patient outcomes [1,2]. Prompt reperfusion therapy, typically achieved through primary percutaneous coronary intervention (PCI), has emerged as the cornerstone of current STEMI management [2]. The critical factor influencing the effectiveness of PCI is the duration between the patient's arrival at the medical facility and the initiation of balloon inflation in the affected coronary artery, termed the door-to-balloon (DTB) time [3].

Over the past decades, substantial efforts have been directed towards minimizing DTB time, recognizing its crucial role in rescuing ischemic myocardium and reducing mortality rates. Guidelines and quality improvement initiatives have emphasized the importance of achieving DTB times within specified benchmarks, often set at 90 minutes or less from the patient's arrival at the catheterization laboratory (Cath lab) [4]. However, achieving timely reperfusion remains a challenge due to various logistical, clinical, and systemic factors [5-7].

Understanding the details of DTB time dynamics and its clinical implications within a specific clinical setting is essential for refining STEMI management protocols, implementing targeted quality improvement measures, and ultimately enhancing patient care and prognosis. So, this observational study aimed to assess DTB times among patients diagnosed with STEMI who undergo primary angioplasty at a tertiary care centre. This study also evaluated the adherence of healthcare providers to recommended DTB time benchmarks while also identifying factors that contribute to delays in the process. This comprehensive analysis sought to offer valuable insights for optimizing DTB times, promoting evidence-based practices, and driving advancements in STEMI patient care.

## Materials and methods

Study design, setting, and duration

This prospective observational study was conducted over 18 months, from March 2017 to August 2018. The research took place at Fortis Hospital Mulund, Mumbai, India, a tertiary care centre equipped with a 300-bed capacity and staffed with a dedicated primary angioplasty in myocardial infarction (PAMI) capable team and cardiac surgery support available round the clock.

Study population and selection criteria

The study population comprised individuals admitted to the Emergency Department of Fortis Hospital Mulund presenting with acute STEMI.

Inclusion and exclusion criteria

This study included individuals aged 18 years and older presenting with chest pain and demonstrating ST elevation on electrocardiogram (ECG), who subsequently underwent PAMI. Patients with non-ST elevation myocardial infarction (NSTEMI), those who received thrombolysis, underwent angioplasty without balloon inflation, rescue angioplasty, or were post-coronary angiography without angioplasty due to medical contraindications such as triple vessel disease, were excluded from the study.

Sample size and sampling

During the study period, a total of 194 patients presented with STEMI. Among them, 15 individuals were identified with triple vessel disease following angiography and were recommended coronary artery bypass grafting (CABG). Additionally, three patients exhibited thrombus presentation post-angiography, while three others underwent thrombolysis. Moreover, two patients opted for medical management. Consequently, the final sample size comprised 171 patients who underwent primary angioplasty. Non-probability sampling was employed for the selection of participants.

Study variables and data analysis

A semi-structured proforma was used for data collection, wherein patient details were extracted from hospital records, while the relevant time intervals were recorded via direct observation. Key time intervals included door-to-ECG time, ECG-to-STEMI activation time, STEMI activation to consent offered time, consent offered to consent obtained time, consent obtained to Cath lab arrival time, and Cath lab arrival to balloon inflation time. Subsequently, data were entered into a Microsoft Excel spreadsheet (Microsoft® Corp., Redmond, United States) and analyzed using SPSS for Windows, Version 15 (Released 2006; SPSS Inc., Chicago, United States). Qualitative data were summarized using frequency and percentages, whereas quantitative data were presented as mean (±SD). T-test was applied to compare the mean duration between the two groups, i.e., DTB time ≤90 minutes and DTB time >90 minutes, and a p-value <0.05 was considered statistically significant.

Ethical consideration

This study was approved by the Institutional Ethics Committee of Fortis Hospital Mulund (IEC/2017/TH/06). Participants were thoroughly briefed about the study. Written consent was obtained from all participants, both for the study itself and for the procedures. All patients received treatment per established guidelines.

## Results

The study sample comprised patients with a mean age of 56.5 (±13.1) years, ranging from 23 to 85 years and predominantly male patients (Table 1). The mean DTB time was 70.21 (±29.16) minutes and the median time was 65.00 (IQR=50-85) minutes, with a substantial proportion of patients achieving DTB times ≤90 minutes, as illustrated in Table 2. Table 3 provides insights into the mean duration required for individual steps constituting the DTB time.

**Table 1:** Baseline characteristics of the patients (n=171)

Variable	Frequency	Percentage
Age group (in years)		
<30	5	7.1%
31 to 40	19	27.0%
41 to 50	24	34.1%
51 to 60	51	72.5%
61 to 70	50	71.1%
71 to 80	18	25.6%
>80	4	5.7%
Gender		
Male	134	78.4%
Female	37	21.6%

**Table 2:** Distribution of door-to-balloon time (n=171)

Time (in minutes)	Frequency	Percentage
≤90	136	79.5%
>90	35	20.5%

**Table 3:** Mean duration of different components of door-to-balloon time (n=171) ECG: Electrocardiogram; STEMI: ST-elevation myocardial infarction; Cath lab: Catheterization laboratory

Door-to-balloon time component	Mean duration (in minutes)
Door to ECG	3.92
ECG to STEMI activation	3.30
STEMI activation to consent offered time	9.42
Consent offered to consent obtained	10.65
Consent obtained to Cath lab arrival time	18.64
Cath lab arrival to balloon inflation	24.37

**Figure 1:**
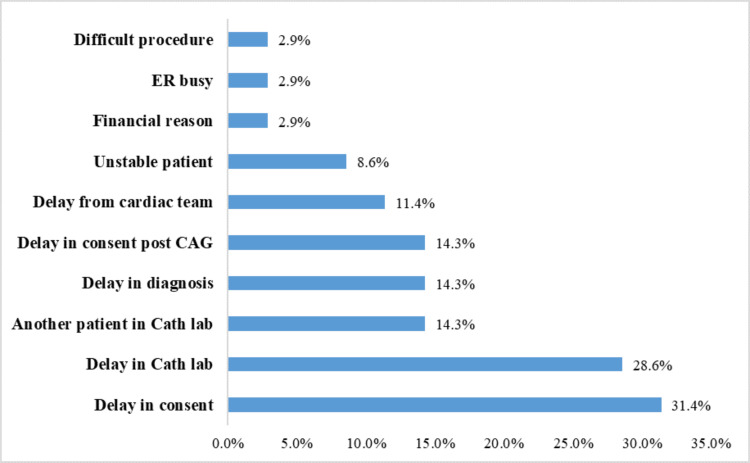
Reasons for delay in door-to-balloon time (multiple reasons) ER: Emergency room; CAG: Coronary angiography; Cath lab: Catheterization laboratory

The observed delays in achieving ≤90 minutes of DTB time in our study were multifactorial, as illustrated in Figure 1. Among these delays, 48.6% (17/35) were attributed to patient-related factors, while the remaining were associated with hospital-related issues. Patient-related delays were predominant, constituting nearly half of the cases. Among these, a significant proportion (31.4%, 11/35) was due to delays in obtaining patient consent. Additionally, delays occurring post-coronary angiography (CAG) consent were noted in 14.3% (5/35) of cases, while 2.9% (1/35) were due to patients not signing financial undertakings promptly. On the other hand, hospital-related delays were predominantly characterized by delays within the Cath lab, representing 28.6% (10/35) of cases, and instances where the Cath lab was occupied by another patient, accounting for 14.3% (5/35) of cases. Other notable hospital-related delays included delays in diagnosis (14.3%; 5/35), as well as delays from the cardiac team (11.4%; 4/35). Furthermore, in 8.6% (3/35) of cases, patients required stabilization of their clinical condition before transfer to the Cath lab. Among the patient subgroups with DTB times exceeding the international cutoff of ≤90 minutes, all procedural steps demonstrated prolonged durations compared to the group with DTB times meeting the specified benchmark, as depicted in Table 4. Notably, while door-to-ECG time exhibited non-significant differences between the two groups, all other intervals were significantly prolonged in the subgroup with DTB times exceeding 90 minutes.

**Table 4:** Comparison of door-to-balloon time component between door-to-balloon time ≤90 minutes vs >90 minutes group ^# ^t-test; * Statistically significant; ECG: Electrocardiogram; STEMI: ST-elevation myocardial infarction; Cath lab: Catheterization laboratory

Door-to-balloon time component	Door-to-balloon time ≤90 min	Door-to-balloon time >90 min	p-value^#^
	Mean (±SD)	Mean (±SD)	
Door to ECG	3.73 (±2.06)	4.71 (±3.56)	0.124
ECG to STEMI activation	2.4 (±3.09)	6.8 (±10.03)	0.000*
STEMI activation to consent offered time	8.04 (±6.10)	14.83 (±13.97)	0.000*
Consent offered to consent obtained	8.4 (±6.63)	19.4 (±20.24)	0.000*
Consent obtained to Cath lab arrival time	15.06 (±11.52)	32.6 (±20.70)	0.000*
Cath lab arrival to balloon inflation	21.27 (±8.71)	36.43 (±18.05)	0.000*

## Discussion

The timely initiation of reperfusion therapy is paramount in the management of STEMI to mitigate myocardial damage and improve clinical outcomes. The DTB time and its association with morbidity and mortality in STEMI management is well established globally. In this study, we sought to determine the DTB time for PAMI and the various time frames influencing the DTB in acute STEMI from a tertiary care hospital.

In this study, the mean age of patients presenting with acute myocardial infarction (MI) was 56.47 years, with a notable predominance of males (78%) compared to females (22%). The highest incidence of acute MI was observed in the age group between 50 and 70 years, with a gradual increase in incidence among females with advancing age, reaching its lowest in those younger than 40 years. The age and gender distribution of STEMI among the participants can be compared to various studies found in the literature. It was observed that the incidence of STEMI was higher among males in comparison to females [1,6-10].

The findings of this study reveal that despite a substantial proportion of patients achieving DTB times within the recommended threshold of ≤90 minutes, a noteworthy subset experienced delays beyond this benchmark. Achieving the international benchmark of DTB time within 90 minutes was attained in a commendable 79.5% of patients. Our institution's well-structured protocols facilitated the minimization of time intervals within the hospital, including door-to-ECG time, activation of the STEMI team, and overall DTB time. Financial undertaking to start the procedure had a positive impact in reducing the DTB time, as this reduced the time taken for consent. Despite a substantial proportion of patients achieving DTB times within the recommended threshold of ≤90 minutes, a noteworthy subset experienced delays beyond this benchmark. The observed delays were multifactorial, with patient-related factors accounting for nearly half of the cases, while hospital-related issues constituted the remainder. Patient-related delays were primarily attributed to procedural hurdles in obtaining consent, both pre-and post-CAG, as well as administrative delays such as the completion of financial undertakings. As seen in the results, significant delays were encountered in obtaining patient consent for the procedure, contributing to 31.4% of cases with a mean consent time of 19.40 minutes. This delay surpassed the standard consent time of 10 minutes set for our study. This pattern of delay is evident from different studies [6,7]. Lack of awareness among patients and their relatives regarding acute MI and its management necessitated extensive discussions, often involving primary physicians or family members, leading to prolonged decision-making processes. This pattern can be seen in other studies done in India, where the lack of awareness of MI among patients and relatives was evident [11-13]. Additionally, financial considerations posed a barrier, particularly among patients without insurance coverage, further impeding the timely initiation of treatment. Conversely, hospital-related delays predominantly stemmed from logistical challenges within the Cath lab and diagnostic processes, further compounded by delays from the cardiac team and the need for patient stabilization before catheterization.

Our study also highlights the association between prolonged DTB times and extended durations in each component of the procedural pathway. Notably, while the door-to-ECG time exhibited non-significant differences between patient subgroups with DTB times ≤90 minutes and >90 minutes, significant disparities were observed in all subsequent intervals. These findings underscore the critical importance of streamlining each step of the DTB process to optimize procedural efficiency and expedite reperfusion therapy initiation.

Efforts to reduce DTB times have been extensively emphasized in contemporary STEMI management guidelines and quality improvement initiatives [3,14-16]. Strategies aimed at enhancing pre-hospital triage, optimizing workflow within the Cath lab, and implementing standardized protocols for consent acquisition are imperative in mitigating delays and improving DTB time adherence. Additionally, leveraging technological advancements, such as telemedicine and pre-hospital ECG transmission, holds promise in facilitating early STEMI recognition and expediting patient transfer to PCI-capable centres. Addressing these multifactorial delays requires a comprehensive approach. Patient education and public awareness initiatives can mitigate delays associated with consent by fostering understanding and expediting decision-making processes. Implementing streamlined financial clearance processes, such as signing financial undertaking forms instead of immediate payment requirements, can alleviate delays due to financial considerations. Improving Cath lab efficiency through 24/7 availability of staff, adequate inventory, and dedicated emergency lab slots can reduce delays attributed to Cath lab unavailability. Empowering emergency departments to directly activate Cath labs without waiting for cardiology consultation can expedite patient care.

Limitations

This study was subject to several limitations that warrant consideration. Firstly, the relatively small sample size may limit the generalizability of the findings to broader patient populations. Additionally, being a single-centre observational study, the results may be susceptible to selection bias, potentially impacting the external validity of our conclusions. The absence of data on the Killip classification, socio-economic status, and the prevalence of pre-existing risk factors such as prior stroke, chronic renal disease, and chronic obstructive pulmonary disease represents a notable limitation. These factors are known to influence patient outcomes in the context of acute MI. Addressing these limitations is crucial for interpreting our findings and underscores the need for larger, multi-centre studies incorporating comprehensive patient data to enhance the strength and generalizability of future research in this field.

## Conclusions

This study highlights the multifaceted challenges in achieving timely DTB times for STEMI patients undergoing primary angioplasty. Addressing these barriers through targeted interventions and systematic protocol enhancements is essential for optimizing STEMI management pathways and improving patient outcomes. By shedding light on the factors influencing DTB delays and their impact on procedural efficiency, our findings offer valuable insights to guide evidence-based strategies aimed at enhancing the timeliness and effectiveness of STEMI care delivery in clinical practice.
